# Enacting healthy checkout policies: lessons from Berkeley and Perris, California

**DOI:** 10.1017/S1368980025101420

**Published:** 2025-11-07

**Authors:** Luc L. Hagenaars, Laura A. Schmidt, Jennifer Falbe

**Affiliations:** 1 Amsterdam UMC Location University of Amsterdam, Department of Public and Occupational Health, Meibergdreef 9, Amsterdam, The Netherlands; 2 University of California San Francisco, School of Medicine, 490 Illinois Street, San Francisco, CA, USA; 3 University of California Davis, Department of Human Ecology, Davis, CA, USA

**Keywords:** Community participation, Policy making, Food industry, Food retail, Impulse buying

## Abstract

**Objective:**

To examine policy processes and industry opposition surrounding the first US healthy checkout ordinances (HCO), which mandate nutritional standards for foods and beverages displayed in grocery checkout areas.

**Design:**

Qualitative case study comparison using Kingdon’s Multiple Streams Framework, triangulating city records, advocacy materials and key informant interviews.

**Setting:**

Local governments of Berkeley and Perris, California, USA.

**Participants:**

Informants, identified from documents and snowball sampling, included community-based organisation members/local advocates (Berkeley *n* 6; Perris *n* 1), staff from national nongovernmental organisations providing assistance (Berkeley *n* 2; Perris *n* 2), city councilmembers (Berkeley *n* 2; Perris *n* 2), city commissioner (Berkeley *n* 1) and city staff (Perris *n* 2).

**Results:**

We described and compared each city’s HCO enactment process. In both, prior commitments to community-led food environment reforms enabled advocates to garner financial and technical support for early coalition building. Berkeley used soda tax proceeds for a youth-led citizen science project to formulate an enforceable HCO and assess public support. These experiences fostered political commitment to define applicable stores, checkout areas and nutritional standards. Campaigns emphasised protecting children and parents from predatory marketing and impulse buying. Berkeley’s campaign quietly and cautiously engaged mostly independent retailers, attracting limited industry attention; Perris engaged all retailers and after enactment faced open opposition from a chain store and trade associations. Perris’ amended HCO included concessions allowing unhealthy items at many endcaps and long checkout lanes.

**Conclusions:**

HCO enactment may be facilitated by prior food policy experience, community capacity, early coalition building, careful policy design and framing and anticipating and managing industry opposition.

Most grocery store checkout lanes are designed to promote impulse purchases of unhealthy snacks and beverages. Recent studies found that 70 % of foods and beverages in store checkouts aisles were unhealthy, and that most price promotions at checkout were for unhealthy foods, defined as sweetened beverages and specific foods containing > 5 g added sugar and > 200 mg Na per serving^(1,2)^. Candy, sweets, sugar-sweetened beverages and salty snacks were common at checkout, whereas water, fruits and vegetables were virtually absent^(1)^. Investigative journalism has uncovered that snack and beverage manufacturers pay retailers ‘slotting fees’ to place their products where consumers are more likely to impulse-buy and that these fees can reach $1 million annually per product in major US chains^(3)^, incentivising unhealthy purchases^(4–6)^, as these fees are primarily affordable to large snack and beverage manufacturers^(3)^.

Studies in the United Kingdom (UK) showed that voluntary standards that restrict unhealthy foods at checkouts can lead to healthier purchases^(7,8)^, but their non-mandatory nature hampered overall effectiveness^(7,9,10)^. In contrast, not a single US chain has voluntarily adopted comprehensive healthy checkout standards. Two local US governments, however, have implemented mandatory healthy checkout policies. Berkeley, CA, implemented the world’s first ‘healthy checkout ordinance’ (HCO) in March 2021^(11)^, followed by a policy in the UK in October 2022, which set nutrition standards for checkouts, aisle endcaps and other prominent locations^(12,13)^. Perris, CA became the second and thus far only other US jurisdiction to implement an HCO in July 2023^(14)^, which was significantly amended directly thereafter^(15)^.

A one-year evaluation of the world’s first healthy checkout policy in Berkeley, CA found a large increase in the healthfulness of checkout food environments and high post-policy compliance (83 % of products)^(16,17)^. An evaluation of Perris’ HCO, which became effective January 2024, is ongoing. Evidence suggests that mandatory healthy checkout policies might contribute positively in multilevel approaches to changing food environments^(6–8,13,16,18–20)^. Because checkout lanes in low-socio-economic status and higher Black and Hispanic composition neighbourhoods are more likely to promote unhealthy foods^(21)^, HCO have the potential to improve health equity^(18)^. HCO can thus positively contribute to public health nutrition strategies, adding to policies such as SSB taxes and front-of-package labeling^(19)^.

While the literature on passing new SSB taxes and front-of-package label policies is well developed^(22–26)^, to our knowledge, no studies have examined the enactment processes of healthy checkout policies. We sought to compare timelines and processes for passing the first U.S. HCO in Berkeley (2021)^(11)^ and Perris (2023)^(14)^. Using key informants interviews, city records and advocacy materials, we analysed differences and similarities between timelines and policy formation in these two cases. Our findings may inform other local US governments considering HCO and are likely applicable to a range of international healthy retail policies (e.g. ‘keep soda in the soda aisle’ policies and comprehensive product placement policies like those in the UK).

## Methods

### Study setting

Berkeley is located in Northern California and has over 124 000 inhabitants, 55 % of which identify as White, 21 % Asian, 11 % Multiracial, 7 % as Black and 12 % Hispanic or Latino. Median household income is moderately higher at $108 558, than $96 334 statewide^(27)^. Adult prevalence of obesity and diabetes is 22·6 % and 6·8 %, respectively^(28)^. All city councilmembers were Democrats during the conduct of this study^(29)^, and the Democratic candidate for the US House won 65·4 % of votes in Berkeley’s voting district in the 2024 election^(30)^. Berkeley houses UC Berkeley and has innovated food environment policies in the last decade including the first US SSB tax. Perris is located in Southern California and has almost 80 000 inhabitants, 79 % of whom identify as Hispanic or Latino, 20 % as White, 3 % as Asian, 17 % Multiracial, and 9 % as Black. Median household income is lower than $82 523 statewide^(31)^. Adult prevalence of obesity and diabetes is 40·8 % and 14 %, respectively^(28)^. All Perris’ city councilmembers were Democrats during our study^(32)^, and the Democratic candidate for the US House won 56·7 % of votes in Perris’ voting district in the 2024 election^(30)^. Perris also innovated food environment policies, such as a 2017 policy that required healthy default beverages with children’s restaurant meals^(33)^.

### Data collection

Data were drawn from city documents and records, advocacy materials and key-informant interviews (Table 1). Documents and records were extracted from the City of Berkeley and City of Perris websites from January to December 2023 and additional pro-HCO advocacy materials requested and received directly from key informants. These correspond to a near-census of documents and records used in the policy adoption process due to the relatively narrow scope of policy and the local setting. During February–May 2023, we interviewed eighteen key informants involved in the enactment of the HCO, who were offered a $50 gift card. Informants were identified through the documents and records and snowball sampling. Lead author LLH conducted these interviews; senior author JF co-interviewed 7/18 informants. Both authors were experienced qualitative researchers. All interviews were held on Zoom, except for 2 in-person interviews held in a location of choice of the interviewee and took in between 30 and 60 min. Informed consent was obtained orally, and interviews were audio-record and transcribed verbatim, with the assurance that recordings and transcripts would not be made publicly available to ensure privacy safeguards. Because of the small census of individuals involved, socio-demographic information were not reported as this could risk identifying participants.

**Table 1.** Documents and records used and key informant affiliations

		Berkeley	Perris
Documents and records	Original and amended ordinances	2	2
	Video recordings of city council meetings where the HCO was discussed	1	4
	Agendas and minutes of city council (both cities) and council subcommittee meetings (Berkeley) where the HCO was discussed	5	6
	Soda tax commission action calendar	1	n/a
	Municipality contracts with community-based organisations	3	0
	City staff report	0	1
	Food industry letters expressing opposition to HCO	2	6
	Pro-HCO advocacy materials used by community-based organisations and local advocates	Miscellaneous and received directly from informants	
Key informants	Community-based organisations and local advocates	6	1
	National and state-level nongovernmental organisations	2	2
	City councilmembers	2	2
	City commissioner	1	n/a
	City staff	0	2

Interview guides were informed by Kingdon’s Multiple Streams Framework (MSF), an established model for understanding the policy formation process (see online supplementary material, Supplemental A)^(34)^. The MSF assumes that policymakers operate under time, knowledge and resource constraints, and that policy problems rarely have straightforward solutions. It defines three separate ‘streams’ flowing into policy formation: (1) the problem stream, where problems gain attention through high-publicity events, changing indicators or policy feedback; (2) the policy stream, where experts, advocates and policymakers develop policy solutions and (3) the political stream, where politicians seek majorities for proposals. A ‘window of opportunity’ for policy change opens when these streams converge due to the strategic efforts by ‘policy entrepreneurs’^(35)^.

Table 1 shows that more Berkeley informants represented community-based organisations (CBO) than in Perris. Informants further included representatives of national and state-level nongovernmental organisations (NGO) that provided funding and technical assistance to both local campaigns. In Perris, one NGO representative played a partial role as a CBO because its representative was active in the city itself. Other informants included city councilmembers, a city commissioner and city staff. Berkeley city staff and the California Grocers Association were invited to participate because informants indicated they were important in Berkeley and Perris, respectively, but did not respond. Perris informants representing a CBO (*n* 1), NGO (*n* 1) and city council (*n* 1) were interviewed again in December 2023 to discuss post-implementation amendments.

### Data analysis

After conducting interviews, we described timelines and processes of HCO policy formation in Berkeley and Perris. Documents and records were used to triangulate factual timeline details provided by informants. We then analysed differences and similarities in these processes thematically, by coding the transcripts in MaxQDA 22.6.1 (VERB) according to the MSF streams, identifying themes related to problem representation, materialisation of the HCO as a policy response and political support. Coding was conducted by the lead author (xx), with the senior author (yy) reviewing a selection of coded transcripts. Documents and records were not coded thematically but served to triangulate information provided by informants. Given the small census of individuals involved, we reached saturation quickly, demonstrated by consistent confirmation of themes across interviews, documents and records. This study was approved as exempt by the UCSF (22-37869) and UC Davis Institutional Review Boards (1794241).

## Results

**Figure 1.**
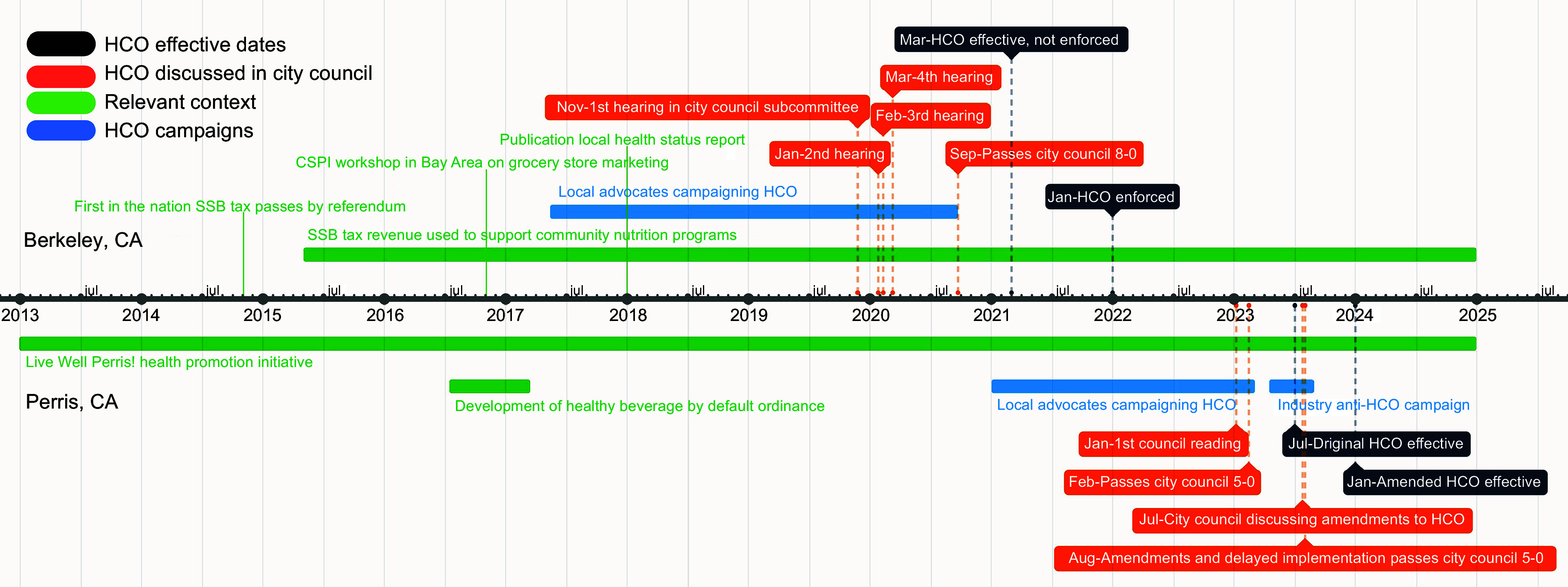
Timelines of events around enactment of healthy checkout ordinances in Berkeley, CA and Perris, CA, 2014–2024.

To compare how Berkeley and Perris enacted their HCO, we first outline the formulation of both cities’ HCO. We then describe timelines (see Figure 1) and streams convergence processes for both cities separately, before presenting thematical differences and similarities in how pro-HCO coalitions acted in the policy and political streams, how the policy stream involved technical challenges in writing the HCO, how industry opposition manifested in the political stream and how strategic framing helped identify the HCO as a solution to the problem of predatory marketing.

### Healthy checkout ordinances formulations in Berkeley and Perris

**Image 1.**
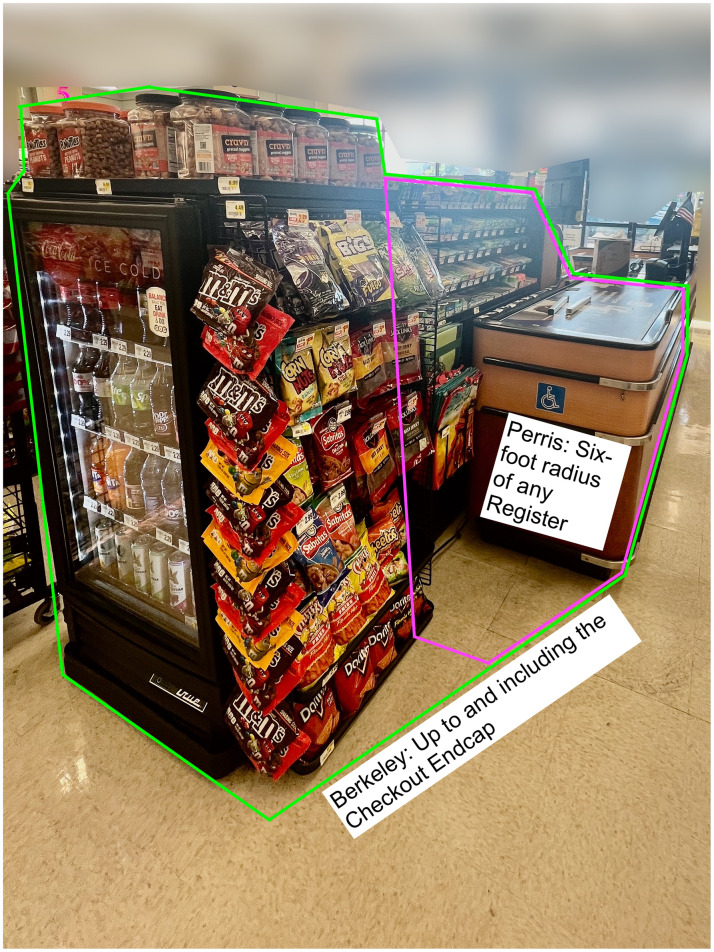
Picture illustrating different definitions of ‘checkout area’ in Berkeley, CA and Perris, CA. The six-foot radius illustrated in pink is an approximation.

Following Kingdon’s MSF, within the policy stream, city officials formulated language for their new HCO. Table 2 outlines how the Berkeley and Perris HCO defined stores, checkout areas and eligible foods and beverages. Both defined eligible stores as those > 2500 sq. ft. that sell food. Perris further limited eligibility to establishments selling groceries including food products and produce, household items and packaged alcohol beverages as an incidental commodity, while excluding small businesses. Berkeley alone adopted the criterion of stores selling ≥ 25 linear ft of food. In practice, this means that Berkeley’s HCO applies to more store types (e.g. drugstores) than Perris’. The definition of checkout also differs. Checkout areas were initially defined in both cities as including areas where customers wait in line, but Perris amended this to include only ‘any area within 6 ft of the cash register’, exempting many endcaps and longer checkout lanes (Image 1).

**Table 2.** Key provisions of Berkeley and Perris healthy checkout ordinances

	Berkeley, adopted 9 September 2020^(11,37)^	Perris original, adopted 14 February 2023^(14)^	Perris after amendments, adopted 29 August 2023^(15)^
Eligible stores	Commercial establishment > 2500 sq ft and ≥ 25 linear ft of food.	Commercial establishment > 2500 sq ft that sells groceries including food products and produce, household items and packaged alcohol beverages as an incidental commodity to the establishment.	Commercial establishment > 2500 sq ft that sells groceries including food products and produce, household items and packaged alcohol beverages as an incidental commodity to the establishment, not including small businesses, with small business defined as ‘an independently owned and operated business that is not dominant in its field of operation with 5 employees or fewer’.
Definition of checkout area	Any area within 3 ft of any register or designated for customers to wait in line to make a purchase, including the checkout endcap.	Any area within 6 ft of any register or an area designated for customers to wait in line to make a purchase.	Any area within 6 ft of any register.
Eligible beverages	Unsweetened beverages.	Water, including carbonated non-caloric water; coffee or tea without added caloric sweeteners (permissible condiments include sugar, sugar substitutes, milk and creamer products); low-fat dairy milk or Ca- and vitamin D-fortified soyamilk with ≤ 200 calories per container; 100 % fruit juice or fruit juice combined with (carbonated) water, with no added caloric sweeteners, ≤ 12 fl oz; 100 % vegetable juice with no added caloric sweeteners, ≤ 300 mg Na per container, ≤ 12 fl oz; beverages with ≤ 40 calories per container.	Water, including carbonated non-caloric water; coffee or tea without added caloric sweeteners (permissible condiments include sugar, sugar substitutes, milk and creamer products); low-fat dairy milk or Ca- and vitamin D-fortified soyamilk with ≤ 200 calories per container; 100 % fruit juice or fruit juice combined with (carbonated) water, with no added caloric sweeteners, ≤ 20 fl oz); 100 % vegetable juice with no added caloric sweeteners, ≤ 300 mg Na per container, ≤ 20 fl oz); beverages with ≤ 40 calories per container.
Eligible foods	Chewing gum and mints without added sugars; fruit, vegetables, nuts, seeds, legumes, yogurt or cheese and whole grains with ≤ 200 mg of Na and ≤ 5 g added sugars per labeled serving.	Food items that contain per package ≤ 200 calories; ≤ 35 % of calories or ≤ 7 g from fat, with the exception of 100 % nuts or seeds; ≤ 10 % of calories or ≤ 2 g from saturated fat, with the exception of 100 % nuts or seeds; 0 g of trans fats; ≤ 35 % of calories or 10 g from total sugars, excepting fruits and vegetables without added sweeteners or fats and yogurts with ≤ 30 g of total sugars per 8 oz container; ≤ 200 mg Na; and meet at least 1 of following standards: consist of sugar-free chewing gum; contain a quarter cup of fruit, non-fried vegetables or fat-free/low-fat dairy; contain 1 oz nuts/seeds or 1 tablespoon of nut butter; contain ≤ 50 % of grain ingredients from whole grain, determined by the product listing whole grain as 1st ingredient or contain 10 % of daily value of Ca, potassium, vitamin D or dietary fibre.	Food items that contain per package ≤ 200 calories; ≤ 35 % of calories or 10 g from total sugars; ≤ 200 mg sodium; Meet at least one of the following standards or have the first ingredient on the list be: sugar-free chewing gum; fruits or vegetables; nuts, seeds, or legumes; whole grains or low-fat or fat-free dairy. The requirements of subsections 1–3 does not apply to fruits, vegetables, nuts, seeds and legumes.

HCO, healthy checkout ordinance.

Nutrition standards also differ. Berkeley’s HCO excludes all sugar-sweetened beverages and artificially sweetened beverages, whereas Perris’s beverage standards are more permissive (allowing low calorie and artificially sweetened beverages) and complex, listing six different categories of permitted beverages each with different limits for calories, sweetener, fat, Na or portion size. Although both cities permit only certain food groups and have nutrient limits, Berkeley limits added sugar and Na per serving, while Perris limits total sugar, Na and calories per package. Thus, applying Perris’ standards (per package) requires separate calculations while applying Berkeley’s does not. Also, in contrast to Berkeley’s nutrient limits, which apply to all permitted foods, Perris exempts fruits, vegetables, nuts, seeds and legumes from nutrient limits. Though still more complex than Berkeley’s standards, Perris’ HCO was amended to simplify its standards by removing limits on fats and minimum servings of certain food groups and nutrients (e.g. fibre, potassium).

### Berkeley healthy checkout ordinance timeline

Participants signalled Berkeley for its progressive stance and history of food policy, most notably, the nation’s first SSB excise tax in 2015, which established a city commission to advise city council on allocating SSB tax revenue to promote public health. Participants and city documents indicated this commission required funded CBO to work on food environment policies within their organisations and the broader community. Also relevant was that Berkeley has few supermarket chains and more locally owned stores.

Participants indicated that Berkeley’s history of food policy made Center for Science in the Public Interest (CSPI), a national consumer advocacy organisation, interested in partnering on healthy retail recommendations^(3)^. CSPI presented these recommendations in a 2016 Bay Area workshop, which identified an HCO as a solution to chronic disease and culminated in the formation of an HCO coalition, thus catalysing the policy stream. Bay Area Community Resources, a youth development CBO, was part of this coalition, and funded by CSPI and the Berkeley soda tax commission to study healthy checkouts. Bay Area Community Resources collaborated with other CBO to conduct citizen science showing that 80 % of checkout options were unhealthy and that 95 % of surveyed residents supported an HCO. CBO and local advocates, but not city staff, expended considerable effort defining applicable stores, checkouts and nutritional standards with guidance from councilmembers.

The HCO was put on the agenda in four Berkeley city council subcommittee meetings between November 2019 and March 2020 (Figure 1). Minutes highlight that two sponsoring councilmembers acted as policy enterpreneurs to converge the three streams. These councilmembers held detailed discussions with the HCO coalition on the research demonstrating the need for and efficacy of healthy checkouts (problem and policy streams), defining eligible products (policy stream) and ways to garner local store support (political stream). After resolving these issues, the HCO was set for enactment by the full city council in April 2020. The COVID-19 pandemic delayed enactment until unanimous approval 22 September 2020. Industry opposition was absent throughout this process. After the city council vote, the city received only a couple of letters from beverage and snack food trade associations requesting that additional products (e.g. artificially sweetened beverages, processed meat) be permitted at checkout.

### Perris healthy checkout ordinance timeline

Participants emphasised that Perris was more ‘working-class’ than Berkeley, that it had a large Hispanic or Latino majority,and that its supermarkets were all local franchises of chains. Participants reported that the city had a dedicated public health division with established relationships with local CBO, which identified local health disparities as a problem among city council. Participants further noted that city officials had worked closely with local CBO and Public Health Advocates, a California-based advocacy group, when innovating one of the nation’s first healthy-beverage-default policies for children’s restaurant meals. Being an innovator and having this network provided momentum for catalysing the problem stream that policy was needed to address health disparities. Perris did not, however, have a local SSB tax or other city funding for CBO working on food systems, as in Berkeley. CSPI did support Public Health Advocates with financial and technical assistance, as it did for Bay Area Community Resources in Berkeley.

In contrast to Berkeley, city council meetings and video recordings highlight that Perris city councilmembers held less detailed discussions about the HCO before enactment, and there were no subcommittee meetings where the HCO was discussed. Public Health Advocates and local CBO presented the idea of an HCO to city council in March and October 2022, after which the city council held a first formal reading on 31 January 2023 followed by its unanimous passage on 14 February 2023. A councilmember mentioned how city staff had extensively consulted with community members and CBO about the HCO’s design, and that in Perris, following Kingdon’s MSF, the policy and political streams converged relatively easily: ‘*When it came out, we were like, “it sounds good…” We do have a lot of trust in our staff.’*

Only after the Perris HCO became effective 1 July 2023 was opposition to the HCO organised in the political stream, as councilmembers received letters critiquing the HCO from the California Grocers Association, another grocery association and an owner of a local chain supermarket. Industry arguments focused on potential revenue and job losses and concerns that the HCO would stop one chain’s development plans, which interviewees perceived as empty threats. According to informants, these critics falsely claimed that the HCO would allow no unhealthy food throughout stores and that nothing could be sold at checkout and conveyed consternation that the policy targeted grocery stores but exempted mass merchandisers, gas stations and pharmacies. Finally, critics charged that Perris’ detailed nutritional standards were confusing.

In response to these critiques, the Perris city council held another meeting on 25 July 2023, attended by the California Grocers Association, local store owners, CSPI and Public Health Advocates. A motion passed 3–2 to delay voting for a motion that would amend the HCO in response to requests for amendments by the California Grocers Association. On 29 August 2023, the Perris City Council passed the amended ordinance unanimously, delaying its effective date to 1 January 2024 (Figure 1). The amendment responded to seven of ten industry requests, including what was perceived by a councilmember as the largest concession – redefining checkout to be ≤ 6 ft of registers: *‘Going down to 6ft does water down the bill, and that’s real. If it had to happen, then it had to happen… It hurt’.*

### Policy and political streams – organisational capacity of pro-healthy checkout ordinance coalitions

Participants emphasised that strong infrastructure of Berkeley and Perris CBO enabled pro-HCO campaigns. This allowed them to build strong arguments about why an HCO was needed (problem stream), what it would look like (policy stream) and to accumulate polling data in both cities demonstrating community support (problem and political streams). The campaign in both cities included petitions, pledges, letters to city council and other opportunities for community members to voice support, such as at council meetings, thereby converging the policy and problem with the political stream. In Berkeley, this was possible through a 2019 amendment to the scope of services of CBO funded through the SSB tax commission. Amendments included that CBO *‘move beyond…education to…engage in…policy, systems, and/or environmental changes’.* Participants emphasised *‘the role of youth was really seminal, talking about [how] they go out and eat in the city, and they’re forced to run in and buy whatever’s easily available’.* In Perris, youth played a less direct role in convincing policymakers, but CBO including those focused on children garnered local commitment in similar ways: *‘I just asked everyone that I know in Perris, “if you support this, can you sign this pledge card?”…. We were … constantly at different community events’*.

The HCO in both cities also benefited from availability of local data, which proved critical for elevating how an HCO could address prevailing issues in the problem stream. Berkeley participants mentioned a timely survey showing policy support, and a councilmember described how ‘*this group of teens who…interviewed people in stores about the potential ordinance*’ convinced council to pursue the HCO. Participants in both cities noted their city’s public health division generated local data on diet-related health disparities. A local health advocate recalled: *‘We were heavily impacted by the first-of-its-kind health impact report, which really looked at the social indicators of health in Berkeley’.*

Participants indicated the City of Berkeley’s public health division was relatively uninvolved, and that the City of Berkeley’s environmental health division expressed concerns about enforcement capacity that were considered during policy formulation. Perris informants mentioned the City of Perris’ public health division had a more proactive role formulating the HCO and reaching out to all stores, after Public Health Advocates and CBO had already done ‘*extensive work…outreaching with the community [and] businesses’.* The city reported having ‘*the luxury of 10 years of history and great relationships and business partners’.*

### Policy stream – technical challenges in writing healthy checkout ordinances

Within the policy stream, participants from both cities elaborately discussed how they defined stores, checkouts, and nutritional standards. A pre-existing threshold Berkeley used for regular inspections explained the decision to only include stores > 2500 sq ft, aligning with advocates’ and councilmembers’ wishes to exclude small ‘*mom-and-pop stores’.* It took multiple in-person store assessments to ensure all Perris stores selling groceries as a large share of their business were included. Initially, for instance, Walmart was excluded, but because Walmart was defined as a grocery store during pandemic lockdowns, the city council included it.

Participants found defining checkout challenging at first. A councilmember mentioned: ‘*If the line snakes through aisles, is it the whole aisle? the people in line that define it? Or is it the distance?…That took up a lot of time’.* Local advocates resolved this problem by taking pictures of various distances from the register and discussing what was acceptable with HCO sponsors. In Berkeley, advocates also worked with healthy-retail-friendly independent grocery stores large enough to be subject to the HCO to ensure the checkout definition was workable, which may have averted opposition. The final definition of the Berkeley HCO included the entire checkout including the endcap, and for stores without a designated checkout area, any space ≤3 ft of the register. However in Perris, where the checkout definition originally included the entire checkout, after initial HCO enactment, a councilmember said, *‘[the California Grocers Association and an opposing local store] wanted 3 feet [from the register only]. At that point, we were like, “why not just make it 6 inches”?’* This led Perris policymakers to re-define checkout as only ≤ 6 feet from the register. Because most checkout endcaps, which contain refrigerators, are > 6 ft, this meant largely excluding sugar-sweetened beverages. A Perris councilmember who expressed regret about this change noted that *‘at least they’re [candy and sodas are] not in the narrow aisle where the kids can grab it’.*

Defining nutritional standards also required substantial effort. Participants sought middle ground between best available science, enforceability and community member preferences. Berkeley prioritised feasibility of enforcement and having strong standards, opting for simpler language. Perris opted for more detailed but permissive standards based in-part on standards for vending machines^(36)^ and community input (Table 2). Participants in both cities mentioned that they adopted stricter language than they might accept, anticipating potential compromises. City staff in Perris mentioned that HCO developers *‘took liberty of putting some extra language in, knowing that we could back off of that’.*

### Political stream – preventing and overcoming food industry opposition

Based on Kingdon’s MSF, food industry actors had an important role in the political stream. While councilmembers, local advocates and NGO all noted that the HCO *‘flew under the radar’* in Berkeley, the Perris HCO drew a decisive response from grocery trade associations and chain store upper management, albeit only after policy enactment. Interviews with councilmembers and local advocates, video recordings and advocacy materials show how the California Grocers Association *‘started contacting all our local grocery stores to try and get letters of opposition’,* leading to what was described as disinformation and exaggeration of the financial impact of the HCO. City officials and councilmembers then sought compromise, eventually leading to seven of ten amendments proposed by the California Grocers Association. Councilmembers ‘*felt like we’re meeting them [California Grocers Association] more than halfway’,* that the amendments ‘*watered down’* the HCO, and that industry would not be satisfied with any level of compromise.

Berkeley and Perris advocates differed regarding which grocery stores they engaged. Advocates and city staff reached out to major chain stores in Perris both before and after HCO enactment, bringing baskets of eligible products to demonstrate feasibility, while city staff held store workshops to prepare implementation. Participants described these actions as garnering support among some store owners, although few lent support during council meetings.

In contrast, Berkeley advocates were more *‘concerned about opposition from big beverage companies*’ and ‘*learned a lot from the soda tax, the science, and…feasibility’,* stating they could not have been the first to pass the HCO *‘without the soda tax’.* This ‘*readiness*’ translated into strategies to ‘*keep this policy intentionally very, very quiet’.* As one noted, *‘when you’re talking to retailers or… councilmembers, you just don’t know who’s working with industry’.* Berkeley advocates *‘did not put anything on social media or op-eds until it went into full council’,* and trade associations did not mobilise prior to enactment. Unlike Perris, Berkeley did not compromise its HCO standards in response to letters sent by beverage and snack food trade associations. These were disregarded by councilmembers as just part of the industry playbook. Berkeley advocates further garnered support from independent stores, about which city council was more concerned, and even chain managers, some of whom had to hold back publicly expressing support because of upper management.

### Problem stream – strategic framing of healthy checkout ordinances

Following Kingdon’s MSF, within the problem stream, all participants explained how the HCO addressed the tangible problem of predatory marketing at checkout, which promoted unhealthy impulse buying, especially among children. According to one councilmember, consumers were *‘totally being manipulated by the whole system all the time’.* A Berkeley advocate recalled that *‘people got it that we need a policy to reduce impulse buying, [that makes] it easier for families and reduced marketing to kids’.* Another Berkeley advocate felt *‘the HCO is there to give people a chance to feel…better about how they interact with the things they purchase, to not feel like they’re being bullied into buying because it’s right there in front of their face’.* A Berkeley councilmember echoed that the HCO addressed the concern that *‘allowing this junk food up at the front forces a terrible choice’.*

Impulse buying also motivated Perris policymakers to uphold the basics of their HCO. According to a Perris councilmember: *‘I have a son, and if he sees a snack at checkout…he’ll grab it. [Industry‘s] argument was it’s a time for educating our kids on healthy choices. I’m not gonna sit there with the line behind me, talking to my toddler about the calorie count’.* Some community members mistakenly thought the HCO would take away choice entirely, which a councilmember rebutted: ‘*It was [important] that everybody understood we’re not taking candy out of the supermarket …. we’re just saying, at the checkout lane, it’s gonna be healthy options’.*

## Discussion

This is the first study to compare the process of enacting first-in-the-nation HCO in two Californian cities, Berkeley and Perris. We found that prior experience with food policy innovations was a key facilitator of HCO success. A history of prior innovation, along with the organisational capacity of local CBO, made both cities attractive partners for technical and financial support from NGO and facilitated community and councilmembers readiness for an HCO. In Berkeley, the city’s soda tax ordinance established community grant making that funded CBO who developed an HCO.

A key barrier for HCO in both cities was the effort required to draft definitions of eligible stores, checkouts,and nutritional standards. Berkeley opted for simple nutritional standards whereas Perris’ standards, especially the original ones, were more complex. Both cities differed in how they engaged stores, with Berkeley focused on engaging locally owned stores interested in healthy options to verify that the policy was workable for stores with fewer resources than chains. Berkeley advocates described how the soda tax campaign taught them to avoid engaging most chains to avoid mobilising industry opposition. In Perris, active outreach to all stores was followed by trade group-orchestrated opposition that limited the scope of the HCO. It is unknown, however, whether opposition in Perris would have occurred regardless of the complexity of its standards or its outreach to chains. For example, industry opposition may have manifested more intensely in Perris due to HCO now being on trade associations’ radars after Berkeley enacted the world’s first HCO.

Finally, organisational capacity was a key factor in HCO passage, which resulted from prior experience with local food policy innovations. In Berkeley, CBO and local advocates – supported by two councilmembers – led most of the work refining the Berkeley HCO. In Perris, city staff collaborated with a CBO in this process. In both cities, advocates framed the HCO as protecting children from marketing and impulse buying. In Berkeley, youth were the face of the HCO campaign, which was perceived by key informants as compelling.

Our results indicate the types of anti-HCO industry arguments that other jursidictions can anticipate. The extent of industry opposition likely depends on which chains are present locally and how a jurisdiction’s stores are connected to well-funded trade associations that represent supermarket chains and soda, candy and snack-food manufacturers. The extent to which pro-HCO advocates are able to anticipate this opposition likely depends on the acceptability of the policy to independent stores and previous experience with industry opposition to food policy innovations. Berkeley’s experience enacting the nation’s first soda tax prepared advocates and city officials for this reality, as evidenced by how the HCO did not attract much industry opposition due to quiet and careful outreach to independent stores, of which Berkeley had more than Perris, and how Berkeley did not compromise the policy. In Perris, local advocates felt they needed to reach out to local chain stores to generate support, but our analysis suggests they were underprepared for the opposition mobilised by the California Grocers Association. This is reflected by the finding that advocates learned, only after the negotiations, that this trade association might not be satisfied with any compromise and that amendments made might have watered down the ordinance too much.

### Strengths and limitations

As the Berkeley and Perris HCO are first-in-the-nation policies, we reached saturation quickly due to interviewing a near census of key informants most involved. Limitations include not interviewing the California Grocers Association, which informants described as influential in Perris, nor Berkeley city staff, despite several unanswered invitations to both. Store owners and manufacturers were not interviewed because our research focused on policy enactment. Implementation research would benefit from including these perspectives. The retrospective qualitative case study design may have resulted in recall errors, as participants have incomplete or selective memories, and snowball sampling may have over-represented certain voices. While industry letters and advocacy materials provided information on the perspectives of trade associations and advocates, these secondary sources may reflect selective reporting and do not capture relevant informal interactions. Triangulation across interviews, records and advocacy materials mitigated some of these risks. Also, because these are only two first-in-the-nation policies in moderately sized California cities with histories of food policy innovation, generalisability to subsequent healthy retail policies in larger jurisdictions may be limited.

### Implications

With Berkeley’s HCO now demonstrating early positive results^(16,17)^, other jurisdictions pursuing healthy retail product placement policies can draw upon both cities’ HCO – especially Berkeley’s simpler and more comprehensive HCO – as a model. Our analysis provides several lessons on enacting HCO. Berkeley and Perris were able to enact their HCO because of early community coalition building, supported by existing networks of CBO experienced in food policy innovations. This underscores the importance for local advocates to develop such networks in advance of pursuing product placement policies and for NGO to support these networks.

Berkeley’s success was due to a local advocacy organisation that stayed quiet until its HCO went to vote, centering youth voices and engaging almost exclusively with small, trusted independent stores to ensure the HCO was straightforward and avoid early industry opposition. This approach could serve as a feasible model for other jurisdictions, emphasising disciplined advocacy and careful outreach to smaller and independent stores.

Furthermore, jurisdictions should consider starting with stricter provisions to allow room for later compromises. Though Berkeley’s HCO remained uncompromised, amendments in Perris suggest that advocates must anticipate that industry trade associations may oppose any restrictions. Advocates should therefore identify non-negotiable elements of an HCO prior to facing opposition. Experiences in Perris further suggest that advocates should be ready to proactively counter disinformation once their HCO goes out to vote, which can mitigate opposition especially when the policy requirements are straightforward.

Finally, other jurisdictions can learn from both cities that HCO-type policies can be effectively and authentically framed around the challenges unhealthy marketing at checkout poses to children and parents, making these policies compelling and easier to communicate.

## Supporting information

Hagenaars et al. supplementary material Hagenaars et al. supplementary material

## Supplementary material

For supplementary material accompanying this paper visit https://doi.org/10.1017/S1368980025101420.
