# Supplementary material for: Enacting healthy checkout policies: lessons from Berkeley and Perris, California
Source: Public Health Nutr. 2025 Nov 7;28(1):e196. doi: 10.1017/S1368980025101420 (PMC12689274; doi:10.1017/S1368980025101420)
Supplement: Hagenaars et al. supplementary material [file S1368980025101420sup001.docx]

**Supplement A. Interview guides for Berkeley and Perris Healthy Checkout Ordinance policy enactment processes**

**Potential Interview Questions for participants involved in Berkeley Healthy Checkout Ordinance**

Start with explanation of the study and who I am (Harkness fellow, just in US for a year to learn about local policies, former athlete, currently health political scientist).

Then the interview commences. It is structured with the following sets of open-ended questions which, depending on the time interviews take and the willingness of participants to respond, may or may not be asked. They will in principle be discussed in the following order.

1. Description of Berkeley Healthy Checkout Ordinance
   1. Can you talk a bit about how the Berkeley Healthy Checkout Ordinance works?
2. Role in agenda-setting and decision-making of Berkeley Healthy Checkout Ordinance
   1. **Can you describe your role in what led to the Berkeley Healthy Checkout Ordinance?**
   2. Can you describe the role of your agency in this process?
3. Factual process of agenda-setting and decision-making of Berkeley Healthy Checkout Ordinance
   1. **Can you describe when and how the idea of enacting this Ordinance was first suggested?**
   2. **Can you describe when and how the decision was made to enact this Ordinance?**
4. Purpose of Berkeley Healthy Checkout Ordinance
   1. What is the purpose or function of the Berkeley Healthy Checkout Ordinance; what problem does it address, in your perspective?
   2. When did you recognize this as a problem? Did you believe it received adequate attention from policymakers up to that point? Why or why not?
   3. **What do you think is the purpose of the Ordinance, according to most other actors/constituents? Is it different than your perception of the Ordinance’s function?**
   4. Were other actors/agencies involved in recognizing it as a problem?
   5. Was problem recognition sudden or incremental? Why?
5. Berkeley Healthy Checkout Ordinance creation
   1. **Was there a particular event or confluent factor that facilitated the Ordinance as a solution to said problem(s)? Why?**
   2. Did you support or oppose the ordinance? What kind of policy did you propose (if any) and why? Was your proposal supported and why?
   3. Did you change your preferences/position to the Ordinance and potentially alternative policies over time? Why and how?
6. **Garnering political support**
   1. Why do you believe the Ordinance received serious attention/found receptive audiences among decision-maker? Other political actors? The general public? When and why?
   2. Do you think interest groups played a role in garnering support? The media? Specific groups, state or national government?
   3. Can you think of any specific factors, not actors, e.g., institutional, environmental, global issue, media reports, that may have contributed toward creating support or opposition to the Ordinance?
7. Leadership
   1. Were there specific individuals who played a big role in pushing for the Ordinance?
   2. Can you tell me how these actors went about gaining support for or generating opposition to the Ordinance?
8. Opposition
   1. **Were there any obstacles to adopting the ordinance?**
   2. **Which entities (if any), opposed the ordinance?**
   3. **Were there any individuals or organizations that were undecided or somewhat opposed to the ordinance that needed to be persuaded? If so, who and how did that persuasion work?**
9. Is there anything else that you think I should ask/may be interested in?
10. Is there any person or organization that you advise me to also interview?

**Potential Interview Questions for participants involved in Perris Healthy Checkout Ordinance**

Start with explanation of the study and who I am (Harkness fellow, just in US for a year to learn about local policies, former athlete, currently health political scientist).

Then the interview commences. It is structured with the following sets of open-ended questions which, depending on the time interviews take and the willingness of participants to respond, may or may not be asked. They will in principle be discussed in the following order.

1. Description of Perris Healthy Checkout Ordinance
   1. **Can you talk a bit about how the Perris Healthy Checkout Ordinance works, technically speaking?**
2. Role in agenda-setting and decision-making of Perris Healthy Checkout Ordinance
   1. **Can you describe your role in what led to the Perris Healthy Checkout Ordinance?**
   2. Can you describe the role of your agency in this process?
3. Factual process of agenda-setting and decision-making of Perris Healthy Checkout Ordinance
   1. **Can you describe when and how the idea of enacting this Ordinance was first suggested?**
   2. Can you describe when and how the decision was made to enact this Ordinance?
4. Purpose of Perris Healthy Checkout Ordinance
   1. **What is the purpose or function of the Perris Healthy Checkout Ordinance; what problem does it address, in your perspective?**
   2. When did you recognize this as a problem? Did you believe it received adequate attention from policymakers up to that point? Why or why not?
   3. **What do you think is the purpose of the Ordinance, according to most other actors/constituents? Is it different than your perception of the Ordinance’s function?**
   4. Was problem recognition sudden or incremental? Why?
5. Perris Healthy Checkout Ordinance creation
   1. **Was there a particular event or confluent factor that facilitated the Ordinance as a solution to said problem(s)? Why?**
      1. **Did the enactment of Healthy checkout policies abroad (eg UK) and domestically (Berkeley) play a role in the ‘birth’ of the idea of going for this ordinance in Perris?**
   2. Did you change your preferences/position to the Ordinance and potentially alternative policies over time? Why and how?
   3. **How did the process of defining Checkout aisles and (non)eligible products look like? Why did the Perris ordinance ended up the way it did?**
   4. **Specifically, why was decided that products need to also contain certain amounts of fruit, vegetables, grains etc, instead the more common way by defining thresholds for grams of sugar, salt and fat?**
   5. **What role did enforcers play in vetting/approving the standards, if any?**
6. **Garnering political support**
   1. Why do you believe the Ordinance received serious attention/found receptive audiences among decision-maker? Other political actors? The general public? When and why?
   2. **What was the role of minority communities and youth?**
   3. **Do you think interest groups played a role in garnering support? The media? Specific groups, state or national government?**
   4. Can you think of any specific factors, not actors, e.g., institutional, environmental, global issue, media reports, that may have contributed toward creating support or opposition to the Ordinance?
7. Leadership
   1. Were there specific individuals who played a big role in pushing for the Ordinance?
   2. Can you tell me how these actors went about gaining support for or generating opposition to the Ordinance?
8. Opposition
   1. Were there any obstacles to adopting the ordinance?
   2. Which entities (if any), opposed the ordinance?
   3. Were there any individuals or organizations that were undecided or somewhat opposed to the ordinance that needed to be persuaded? If so, who and how did that persuasion work?
9. Implementation – future expectations

The ordinance got accepted feb 14, 2023, and is set to be implemented on July1.

- 1. **Are retailers ready for implementation?**
  2. **Do you expect any teething or more major problems to implement this ordinance?**

1. Is there anything else that you think I should ask/may be interested in?
2. Is there any person or organization that you advise me to also interview?
